# Supplementary material for: Pollen Proteases Play Multiple Roles in Allergic Disorders
Source: Int J Mol Sci. 2020 May 19;21(10):3578. doi: 10.3390/ijms21103578 (PMC7278992; doi:10.3390/ijms21103578)
Supplement: Supplementary file 1 [file ijms-21-03578-s001.pdf]

Supplementary Information

Results

Proteolytic profile of the pollen diffusates

The pollen extracts of *C. album*, *E. globulus* and *P. lanceolata* were analyzed by SDS-PAGE in order to obtain a protein profile in terms of molecular mass. The quantity of total protein released was superior in pollen extracts with low allergenicity and larger pollen grain dimensions, approximately 0.75 ± 0.18 mg/ml for *E. globulus*. While, for *C. album* and *P. lanceolata* with moderate allergenic potential and small dimensions of their pollen grains, had an average of total protein released of (0.26 ± 0.06 mg/ml and 0.18 ± 0.03 mg/ml, respectively. In order to enhance the sensitivity of visual identification of the protein profile, it was necessary to resort to staining with Silver Nitrate, more sensitive then Coomassie Blue (Figure SI1).

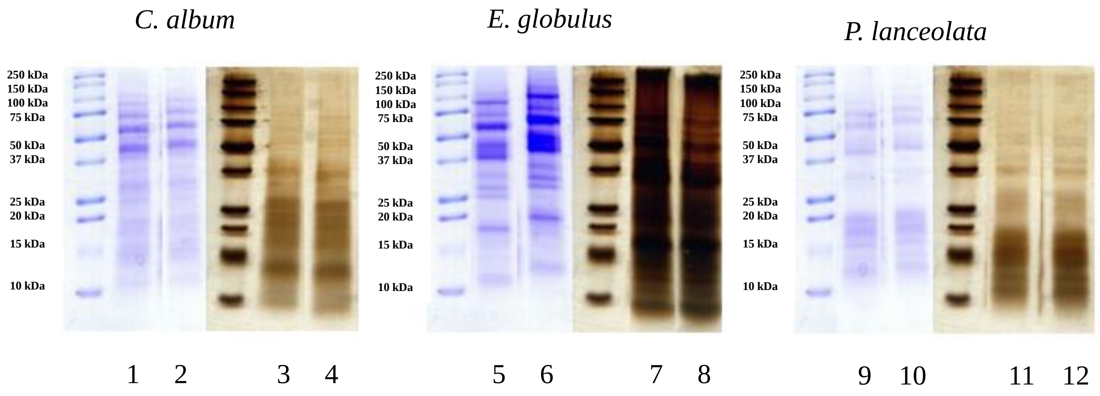

Figure SI1. Protein profile of the pollen extracts in 12.5% SDS-PAGE gels. Staining was performed with Coomassie Blue, first profiles and with silver nitrate, last profiles, for each pollen extract. **1 and 3)** 3.5 µg of pollen extract from *C. album*. **2 and 4)** 5 µg of pollen extract from *C. album*. **5 and 7)** 8 µg of pollen extract from *E. globulus*. **6 and 8)** 12 µg of pollen extract from *E. globulus*. **9 and 11)** 2 µg of extract from *P. lanceolata*. **10 and 12)** 3 µg of pollen extract from *P. lanceolata*.

The presence of proteases in these pollens diffusates was assessed by gelatin zymography. Zymography is an enzymatic assay which uses gelatin as a substrate co-polymerized in an SDS-PAGE gel. Gelatin is used, since it is derived from collagen and for that reason efficient in the detection of a range of proteases.

For the identification of proteolytic activity in the pollen extracts, these were prepared in non-denaturing conditions in order to preserve the enzymatic activity, and subjected to an electrophoresis in a polyacrylamide gel containing 1 mg/ml gelatin. After the electrophoretical separation, SDS was removed using Triton X-100, incubated overnight at 37 ºC and in buffer pH 7.4. Finally staining with Coomassie Blue allowed the detection of enzymatic digestion spots that corresponded to non-colored bands (Figure SI2).

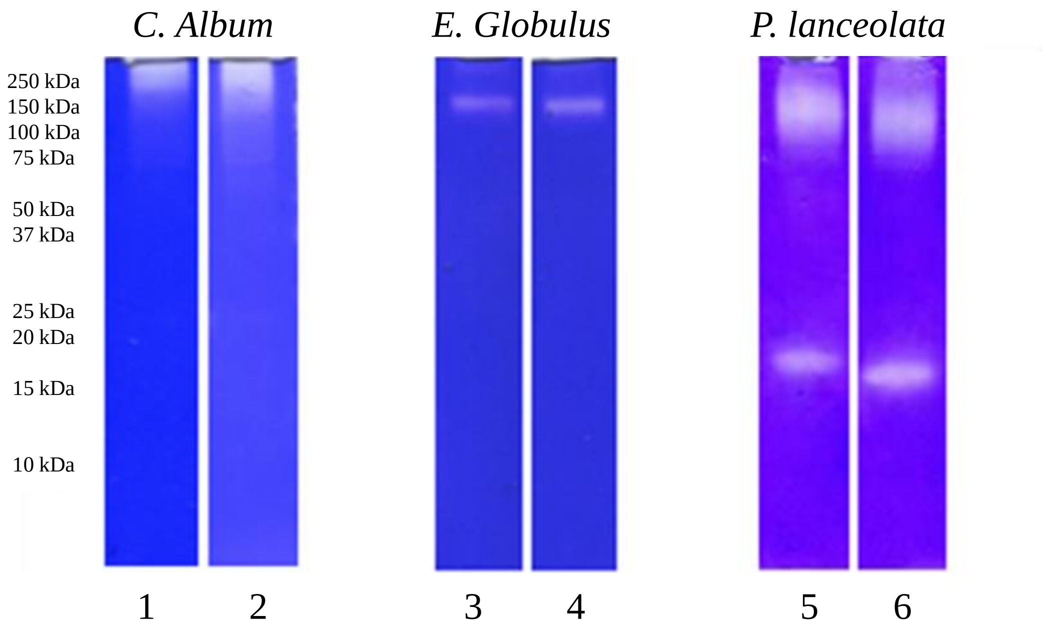

Figure SI2. Proteolytic profiles of pollen extracts obtain by zymography 12% polyacrylamide co-polymerized with 1 mg/ml gelatin. **1)** 3.5 µg of the initial pollen extract from *C. album.* **2)** 5 µg of the initial pollen extract from *C. album.* **3)** 8 µg of the initial pollen extract from *E. globulus*. **4)** 12 µg of the initial pollen extract from *E. globulus*. **5)** 2 µg of the initial pollen extract from *P. lanceolata*. **6)** 3 µg of the initial pollen extract from *P. lanceolata*.

Additionally, pollen proteolytic activity was rapidly characterized through two-dimensional zymography. For first dimension, concentrated pollen extracts samples were submitted to an isoelectric focusing, using pH 3-10 strips, were separation occurred accordingly to their pI. The second dimension, separation occurs accordingly to their molecular mass, on a gelatin zymography. Revelation of enzymatic digested spots was possible through Coomassie Blue staining (Figure SI3).

Pollen extracts samples were concentrated using centricon filter devices of 30kDa, in order to increase the total quantity of protein. This method intends to remove proteins of low molecular weight and concentrate the samples of pollen extracts in high molecular weight proteins, region normally comprised of the majority of proteases.

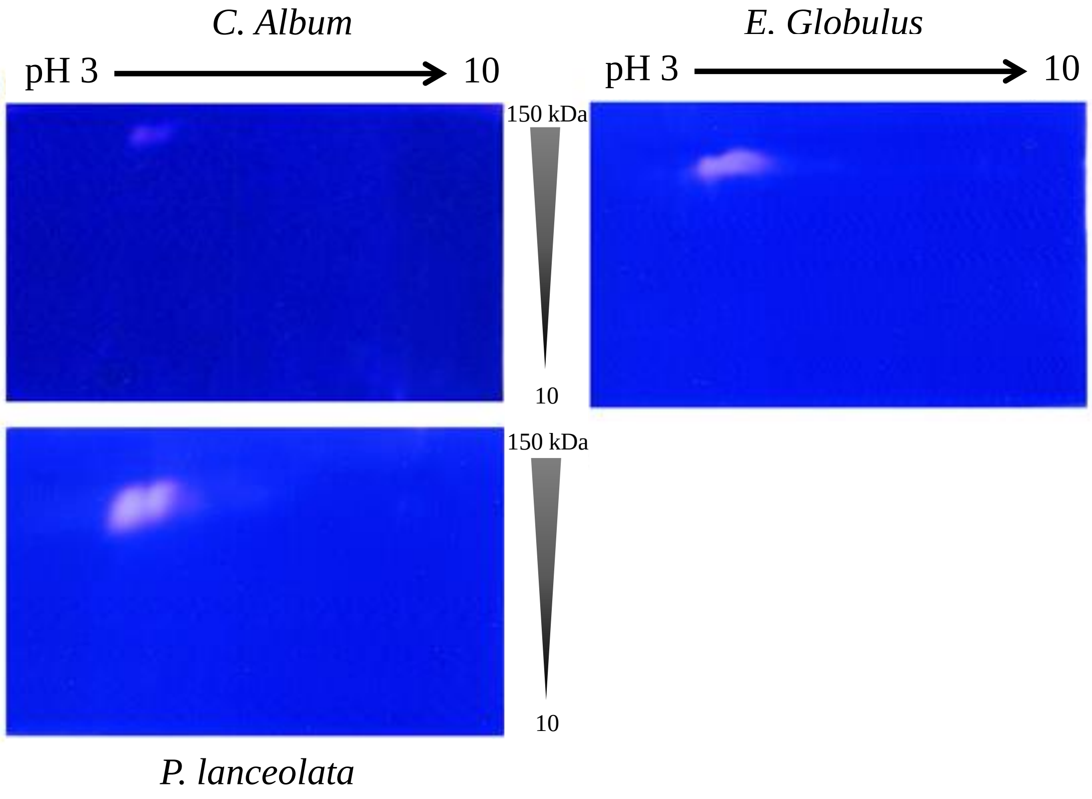

Figure SI3. Proteolytic 2D-PAGE profile of the concentrated pollen extracts of *C. album*, *E. globulus* and *P. lanceolata.* The total quantity of each sample of concentrated pollen extract was ~100 µg.

In conclusion, it was shown that all pollen diffusates contained gelatin degrading proteases which are of high molecular weight and acidic pI (Figure SI1-3).

Effect of pollen diffusates on intercellular adhesion protein integrity

The effect of pollen diffusates on intercellular protein complexes was evaluated by immunocytochemistry. Microscopy detection of disrupted protein junctions could be easily observed by evident interruptions in the continuous ring at the epithelial cell apices, caused by the disassembly of macromolecular protein complexes. As expected all pollen diffusates induced interruptions in the

epithelial cell apices when labeled for E-cadherin, claudin-1, occludin and ZO-1 (Figure SI4). Noteworthy, is the fact that *C. album* was used in this assay at a 1:20 dilution as this pollen diffusate induced a high degree of cell detachment.

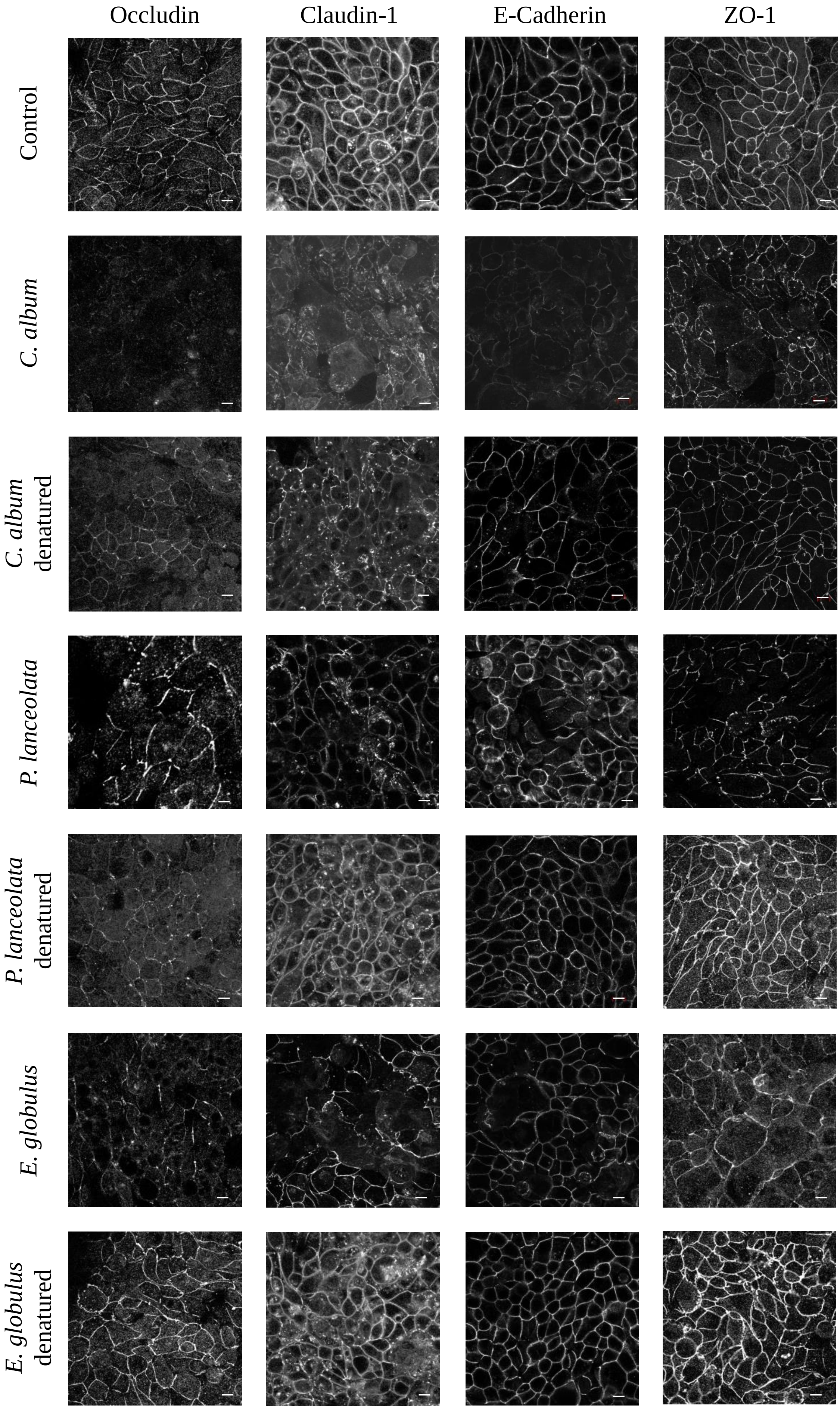
Occludin
Claudin-1
E-Cadherin
ZO-1
Control
C. album
C. album denatured
P. lanceolata
P. lanceolata denatured
E. globulus
E. globulus denatured

Figure SI4. Effect of pollen diffusates on intercellular adhesion protein integrity analyzed using an immunofluorescence assay. Calu-3 cells were incubated with pollen diffusates from *Chenopodium album* (0.013 ± 0.01 mg/ml), *Plantago lanceolata* (0.18 ± 0.03 mg/ml) and *Eucalyptus globulus* pollen diffusates (0.75 ± 0.18 mg/ml) for 6 h. The cells were also exposed to denatured pollen diffusates (95 ºC for 30 min). Cells incubated with culture medium were used as a control. Representative images are shown for each stimulus. Image scale bar corresponds to 10 μm. Imaging to detect unspecific labelling was also performed and such labeling was shown to be non-existent (images not shown).

Material and Methods

Immunocytochemistry

Calu-3 cells were washed with PBS and incubated for 6 h with pollen diffusates diluted 1:1 with serum-free culture medium. Following incubation, cells were washed twice with PBS and fixed with methanol at -20 ºC for 20 min. Cells were permeabilized with 0.3 % (v/v) Triton X-100 in PBS at 4 ºC for 10 min. To block nonspecific labelling cells were incubated with 10 % (m/v) BSA in PBS-T (PBS with 0.1 % (v/v) Tween 20) for 1 h at room temperature. Cells were then incubated with primary antibodies diluted in 10 % (m/v) BSA in PBS-T for 48 h at 4 °C. The following primary antibodies were used: mouse monoclonal anti-occludin (1:300), rabbit polyclonal anti-claudin-1 (1:100), mouse monoclonal anti-ZO1 (1:100) (Zymed Laboratories, Barcelona, Spain) and mouse monoclonal anti-E-cadherin (1:100) (BD Biosciences, Franklin Lakes, USA). Cells were then washed five times with PBS solution containing 0.1 % Tween and 0.1 % gelatin and incubated with the appropriate secondary antibodies (Alexa Flour 488 rabbit (1:1000) and 543 mouse (1:1000) (Invitrogen)) for 1 h at room temperature. The fluorescent dye Hoechst 33342 (Sigma, 0.5 μg/ml) was used to stain nuclei. The transwell membrane inserts were cut and mounted on a fluorescent mounting medium (DAKO, Denmark A/S, Denmark) and imaging performed using an

LSM 510 Meta confocal microscope (Carl Zeiss) with an ApoChromat 63×/1.4 oil objective using the lasers Argon 2 (488 nm), DPSS (561 nm) and Drode (405 nm).
